# Microbial Metazoa Are Microbes Too

**DOI:** 10.1128/mSystems.00109-19

**Published:** 2019-06-04

**Authors:** Holly M. Bik

**Affiliations:** a Department of Nematology, University of California, Riverside, Riverside, California, USA

**Keywords:** early career researcher, marine sediments, microbial metazoa, microbiome, symbioses, terrestrial soils

## Abstract

Microbial metazoa inhabit a certain “Goldilocks zone,” where conditions are *just right* for the continued ignorance of these taxa. These microscopic animal species have body sizes of <1 mm and include diverse groups such as nematodes, tardigrades, kinorhynchs, loriciferans, and platyhelminths. The majority of species are *too large* to be considered in single-cell genomics approaches, yet *too small* to be wrapped into international genome sequencing initiatives. Other microbial eukaryote groups (namely the fungal and protist communities) have gained significant momentum in recent years, driven by a strong community of researchers united behind a common goal of culturing and sequencing new representatives. However, due to historical factors and difficult taxonomy, persistent research silos still exist for most microbial metazoan groups, and public molecular databases remain sparsely populated. Here, I argue that small metazoa should be embraced as a key component of microbial ecology studies, promoting a holistic and cutting-edge view of natural ecosystems.

## Abstract (precis)

Microbial metazoa inhabit a certain “Goldilocks zone,” where conditions are *just right* for the continued ignorance of these taxa. These microscopic animal species have body sizes of <1 mm and include diverse groups such as nematodes, tardigrades, kinorhynchs, loriciferans, and platyhelminths.

## PERSPECTIVE

What is a “microbe”? For some researchers, this term has a very narrow definition, including only prokaryotic taxa within the domains of *Bacteria* and *Archaea*. But from an environmental viewpoint—one that takes into account species interactions and ecosystem function—the gamut of microbes must include a much wider biological spectrum, inclusive of viruses, unicellular and colonial eukaryotes, microbial animal phyla, and eggs and larval stages of larger vertebrate and invertebrate species. Perhaps the least amount of research effort is currently devoted to microbial metazoan species—abundant and ubiquitous taxa that are poorly sampled and scarcely understood.

**FIG 1:**
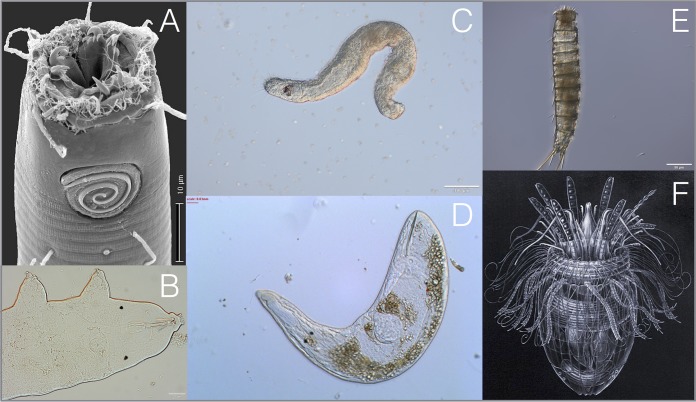
Microbial metazoa, often collectively referred to as “meiofauna,” include species from >20 phyla, such as nematodes (A), tardigrades (B), platyhelminths (D), and kinorhynchs (E), as well as recently discovered, enigmatic groups such as the *Gnathostomulida* (C) and *Loricifera* (F). Nematode image courtesy of Jim Baldwin and Manuel Mundo-Ocampo at University of California, Riverside; tardigrade, kinorhynch, and *Gnathostomulida* images courtesy of Kevin Kocot and Rebecca Varney at the University of Alabama; all other images are CC-licensed and obtained from Wikipedia (*Loricifera*) and Encyclopedia of Life (platyhelminths).

Microbial metazoa are operationally defined as multicellular eukaryote species within the Animalia exhibiting a body size of <1 mm in length. In soil and marine ecology, these taxa are collectively referred to as the “meiofauna,” which includes all organisms retained on a 38- to 45-μm sieve but that pass through larger mesh sizes. Thus, meiofaunal taxa include phylogenetically divergent but poorly characterized groups such as nematodes, tardigrades, gastrotrichs, copepods, kinorhynchs, platyhelminths, and rotifers. Microbial metazoan species can be found across >20 animal phyla, including recently discovered and enigmatic lineages such as the *Gnathostomulida* and *Loricifera* (Fig. 1). Groups such as nematodes exhibit high biodiversity, rampant cryptic speciation, and impressive densities (up to 84 million individuals per m^2^ in estuarine mud [1]), yet for most lineages, we lack even basic genomic data and molecular phylogenies. For example, no published genome sequence exists for any free-living marine nematode species, and molecular data for most cosmopolitan genera are restricted to a handful of short 18S rRNA gene barcodes in GenBank.

Sparse metazoan database resources are depressingly common. Microbial metazoan taxa represent <15% of published or in-progress genome sequences listed in the Joint Genome Institute’s GOLD database (https://gold.jgi.doe.gov/). In the case of nematodes, available genome data sets are overwhelmingly focused on parasitic taxa (*Ascaris*, *Brugia*, *Globodera*, etc.) as well as Caenorhabditis elegans models and their close phylogenetic relatives. Even rRNA gene databases are far from adequate: the latest release of the SILVA database reports only 25,511 sequences for 18S rRNA genes with taxonomic assignments to metazoan species, in contrast to 592,561 and 25,026 entries for 16S rRNA genes from bacteria and archaea, respectively (SILVA release v132, nonredundant SSURef [https://www.arb-silva.de/]). The poor quality of metazoan reference databases makes it difficult to derive robust taxonomy assignments from environmental metabarcoding data (even when using the “best” option of rRNA marker genes) and precludes useful eukaryotic gene annotations generated from environmental metagenomes or single-organism genome sequences.

Why haven’t metazoan species been embraced by the field of microbial ecology? These small animal phyla are often excluded from environmental sequencing studies due to metabarcoding primer choices and study designs that do not adequately capture metazoa. For example, many eukaryotic metabarcoding primer sets (e.g., amplifying 18S rRNA and mitochondrial cytochrome *c* oxidase 1 genes) are targeted toward the recovery of single-cell protist lineages or larger animals (macrofaunal invertebrates and bony fish [2]). Furthermore, microbial ecology DNA extractions using the typical input of 2.5 g of bulk soil/sediment will recover very few metazoan taxa, and much larger sample volumes (>100 g [3]) or labor-intensive sample-processing steps (e.g., decanting and sieving [4]) are required for robust metazoan community assessments.

Although microbial animal species are large enough to be physically manipulated, their minute size makes them too small to be considered for most microbiome studies and genome sequencing efforts. Taxonomic expertise is needed to identify and isolate organisms, and molecular work often requires customized lab protocols optimized for low inputs of DNA. While some metazoan-focused studies are able to pool multiple organisms together to increase biomass and DNA quantity (e.g., studies of the C. elegans microbiome, where 30 to 100 worms per species/treatment are typically pooled prior to DNA extraction [5]), the majority of metazoan-focused investigations require single-organism sequencing approaches. High biodiversity and unclear species boundaries are typical of speciose groups such as nematodes (6), meaning that two specimens with similar morphology are never guaranteed to be conspecifics.

Combined with longstanding taxonomic deficits across many animal phyla (many species observed but few formally described), the above factors effectively place microbial metazoa into a “Goldilocks zone of neglect.” This continued ignorance stems from historical factors and practical constraints, not biological irrelevance. Indeed, the artificial neglect of small metazoan species is unfounded and increasingly alarming in the face of climate change impacts and worldwide habitat loss. Research in my lab strongly emphasizes that microbial metazoa are microbes too. Our ongoing projects are aiming to (i) generate baseline data on ecological and evolutionary patterns in microbial metazoa (and their associated microbiomes) across diverse habitats and geographic regions, (ii) develop wet lab protocols and bioinformatics workflows that facilitate easier incorporation of metazoan taxa into microbial ecology studies, and (iii) build a global community of interdisciplinary researchers who can help solve longstanding scientific challenges (e.g., poor taxonomy and an ongoing lack of metazoan genome resources). Two exciting research directions focus on patterns in nematode microbiomes and the use of environmental -omics surveys for rapid biomonitoring of marine sediment habitats.

For microbial metazoan taxa, we are briskly acknowledging the importance of bacterial symbioses and host-associated microbial assemblages. The study of nematode microbiomes represents one such emerging research area, encompassing lab-based studies of model organisms and cultured species (e.g., C. elegans [5]), as well as exploratory sequencing of worms isolated directly from natural environments (7–8). Nematode microbiomes have been shown to comprise lower-diversity community assemblages that are distinct from the overall mixture of microbes present in the surrounding soil or sediment (5, 7). Further evidence suggests strong environmental filtering and evolutionary conservation of host-associated taxa: “natural” microbiome profiles can be restored to gnotobiotic C. elegans strains (5), and distinct microbiome assemblages appear to be correlated with resource partitioning and increased tolerance to temperature/salinity ranges in cryptic species complexes of *Litoditis* marine nematodes (8). Microbiome taxa have also been shown to confer pathogen resistance and host survival during environmental stress, and external bacterial cues are thought to play a key role in governing the reproductive timing of C. elegans populations on rotting fruit (5). In marine ecosystems, *Astomonema* spp. and Stilbonematidae nematodes are known to carry dense loads of sulfur-utilizing bacterial symbionts, with recent investigations demonstrating that some ectosymbionts of *Laxus* nematodes are even capable of nitrogen fixation (9). Thus, the most compelling questions for metazoan microbiome studies focus not only on the characterization of the microbes themselves (how and why prokaryotic taxa adopt a host-associated lifestyle) but also on the consequences for host evolution and speciation (how microbiome taxa impact host fitness, tolerance to abiotic factors, and niche partitioning).

Metazoan taxa must also be incorporated into large biological surveys of soils and sediments, including both high-throughput -omics projects and traditional (taxonomic) efforts to characterize biodiversity and habitat status. To date, metazoan species have been largely excluded from prominent microbial ecology initiatives such as the Earth Microbiome Project (http://earthmicrobiome.org/), NEON (https://www.neonscience.org/), and TARA Oceans (https://oceans.taraexpeditions.org/). These expansive projects represent global efforts to characterize microbial biodiversity and species distributions using high-throughput marker gene sequencing and metagenomics. However, eukaryotic assessments have been restricted to fungi and protist lineages, and metazoan taxa (if recovered during sequencing) are largely excluded from downstream data analyses. In contrast, metazoan species have been embraced by “Biomonitoring 2.0” initiatives (10) that focus on improving aquatic and marine ecosystem assessments via incorporation of environmental DNA (eDNA) capture and high-throughput marker gene sequencing. Such biomonitoring projects aim to improve the speed and biological resolution of traditional survey techniques that are used by environmental managers to determine habitat status and mitigation/restoration needs (e.g., following input of chemical pollutants or physical habitat disturbance). While this is a notable goal and much progress has been made in developing technical protocols, the major Biomonitoring 2.0 initiatives are overwhelmingly focused on detection of large macroinvertebrate species (insects, molluscs, and crustaceans) and vertebrates (fish and marine mammals). Parallel assessments of smaller meiofauna species would provide an even deeper view of ecosystem function. Groups such as nematodes have poor dispersal capabilities, short generation times, and well-documented responses to pollutants and ecological stress (11). In addition, hyperdiverse meiofauna assemblages facilitate the identification of specific bioindicator taxa, whereby some species are highly sensitive to disturbance impacts (e.g., kinorhynchs [12]), while other species can persist even in the most heavily impacted environments (predatory nematodes following oil spills [13]). Characterization of microbial metazoan assemblages will be critical for gaining a holistic understanding of ecosystem function, as well as assessing habitat resilience and recovery following major disturbance events.

The global importance of microbial metazoa cannot be understated. The abundance, diversity, and ubiquity of these small animal phyla hint at their important (but as yet undescribed) roles in ecosystem function and nutrient cycling. Microbial ecology studies will gain immense benefits from incorporating broader assessments of microbial metazoan species, facilitating rapid biodiversity discovery and generation of sorely needed public genome resources. Historical research silos should no longer exist in an age of integrated -omics capabilities. With any luck, conditions are *just right* for the scientific community to embrace small metazoan taxa as integral components of microbial communities.
